# m^6^A regulators featured by tumor immune microenvironment landscapes and correlated with immunotherapy in non-small cell lung cancer (NSCLC)

**DOI:** 10.3389/fonc.2022.1087753

**Published:** 2022-12-16

**Authors:** Baowen Yuan, Hao Qin, Jingyao Zhang, Min Zhang, Yunkai Yang, Xu Teng, Hefen Yu, Wei Huang, Yan Wang

**Affiliations:** ^1^ Key Laboratory of Cancer and Microbiome, State Key Laboratory of Molecular Oncology, National Cancer Center/National Clinical Research Center for Cancer/Cancer Hospital, Chinese Academy of Medical Sciences and Peking Union Medical College, Beijing, China; ^2^ Beijing Key Laboratory of Cancer Invasion and Metastasis Research, Department of Biochemistry and Molecular Biology, School of Basic Medical Sciences, Capital Medical University, Beijing, China

**Keywords:** m^6^A regulators, m^6^A modification, tumor immune microenvironment (TME), immunotherapy, non-small cell lung cancer (NSCLC)

## Abstract

**Introduction:**

Recent research has confirmed the critical role that epigenetic factors play in regulating the immune response. Nonetheless, what role m^6^A methylation modification might play in the immune response of non-small cell lung cancer (NSCLC) remains vague.

**Methods:**

Herein, the gene expression, copy number variations (CNVs), and somatic mutations of 31 m^6^A regulators in NSCLC and adjacent control samples from the GEO and TCGA databases were comprehensively explored. Using consensus clustering, m^6^A modification patterns were identified. Correlations between m^6^A modification patterns and immune cell infiltration traits in the tumor immune microenvironment (TME) were systematically analyzed. Differentially expressed genes were verified and screened by random forest and cox regression analysis by comparing different m^6^A modification patterns. Based on the retained gene panel, a risk model was built, and m^6^Ascore for each sample was calculated. The function of m^6^Ascore in NSCLC prognosis, tumor somatic mutations, and chemotherapy/immunotherapy response prediction were evaluated.

**Results:**

Consensus clustering classified all NSCLC samples into two m^6^A clusters (m^6^A_clusterA and m^6^A_clusterB) according to the expression levels of 25 m^6^A regulator genes. Hierarchical clustering further divides the NSCLC samples into two m^6^A gene clusters: m^6^AgeneclusterA and m^6^AgeneclusterB. A panel of 83 genes was screened from the 194 differentially expressed genes between m^6^A gene clusters. Based on this, a risk score model was established. m^6^A modification clusters, m^6^A gene clusters, and m^6^Ascore calculated from the risk model were able to predict tumor stages, immune cell infiltration, clinical prognosis, and tumor somatic mutations. NSCLC patients with high m^6^Ascore have poor drug resistance to chemotherapy drugs (Cisplatin and Gemcitabine) and exhibit considerable therapeutic benefits and favorable clinical responses to anti-PD1 or anti-CTLA4 immunotherapy.

**Discussion:**

In conclusion, methylation modification patterns mediated by the m^6^A regulators in individuals play a non-negligible role in prognosis prediction and immunotherapy response, which will facilitate personalized treatment and immunotherapeutic strategies for NSCLC patients in the future.

## Introduction

As the second most frequent malignant oncologic disease worldwide, lung cancer accounts for the greatest number of mortality (1). 85% of lung cancers are non-small cell lung cancers (NSCLC). Due to the majority of NSCLC patients being diagnosed at an advanced stage, its overall 5-year survival rate is only 8% (2). Therefore, new approaches are urgently required to explore novel mechanisms of NSCLC that are susceptible to therapeutic inventions. Recent research has shown that tumor immune microenvironment (TME) immune cell infiltrating characteristics are closely correlated with m^6^A modifications, which might provide an alternative choice (3–5). N6-methyladenosine (m^6^A) RNA modification is the most prevalent kind of RNA modification in eukaryotic cells. It is essential for the regulation of epigenetic processes, a variety of physiological functions, and the development of disease (4, 6, 7).

Methyltransferases, also known as “writers”, promote m^6^A methylation modification in RNA; demethylases, also known as “erasers”, remove m^6^A methyl groups from RNA; and binding proteins, also known as “readers”, bind to the m^6^A methylation site in RNA and perform specific biological functions. Three types of proteins regulate the dynamic and reversible process of m^6^A modification (7, 8). Comprehending the functions of m^6^A modification in post-transcriptional regulation would be more accessible by comprehensively exploring the expression and function of m^6^A regulatory proteins (9, 10). The development of malignant tumors and immunomodulatory disorders are correlated with dysregulated expression and genetic alterations of m^6^A regulator genes (10–12), demonstrating that m^6^A regulators may be crucial in regulating the immunological microenvironment of malignancies.

An increasing number of research have revealed the relationships between m^6^A modifications and the immune cell infiltrating characteristics of the TME (3–5). Wang et al. reported that dendritic cell activation and maturation were aided by METTL3-mediated m^6^A modification. Co-stimulatory molecules CD80 and CD40 are expressed less when METTL3 is knocked out (13). Studies suggest the vital role of TME in cancer progression and therapeutic responses with increasing evidence (14, 15). The immune response and the benefit of chemotherapy are reflected in the TME context that was established at diagnosis (15, 16). Clinical outcomes in various cancers are correlated with changes in the compositions of CD8 and CD4 positive T cells, macrophages, and cancer-associated fibroblast infiltration in the TME (17, 18).

The immune checkpoint blockade (ICB) therapy, which specifically targets the cytotoxic T lymphocyte antigen 4 (CTLA-4) and programmed cell death 1 (PD-1) or its ligands (PD-L1), has been used for cancer immunotherapy and has shown promising clinical results (19). Only a small portion of patients, nevertheless, might benefit from ICB treatment. Thus, exploring the TME and its associated mechanisms is urgently needed to improve immunotherapy’s efficacy. As was previously stated, the microenvironment of malignancies and immune cells are closely correlated with m^6^A modifications. Therefore, our comprehension of immunological regulation in the TME and immunotherapeutic tactics development will be enhanced by fully exploiting the effects of the regulatory network of RNA m^6^A modification enzymes on TME cells.

By thoroughly analyzing the gene expression profile of m^6^A regulators in 1558 NSCLC samples, the present study could distinguish different m^6^A modification patterns. We systematically correlated the characteristics of TME cell infiltration with genomic traits as well as the clinical and pathologic characteristics of NSCLC. We estimated the patterns of TME infiltration in 1,558 NSCLC samples. Further, a risk model was constructed based on a panel of 83 genes with differential expression, and m^6^Ascore was calculated for each sample. Consequently, we developed a method for quantifying the m^6^Ascore and found that it is a robust prognostic biomarker and a significant predictor of response to chemotherapy (Cisplatin and Gemcitabine) and immunotherapy (anti-PD1 or anti-CTLA4 immunotherapy).

## Materials and methods

### Data collection and pre-processing

The study’s workflow is depicted in **Figure S1**. TCGA Gene expression data, genomic mutation data (including somatic mutation and copy number variation), and corresponding clinical data of NSCLC samples were downloaded from UCSC Xena (https://xenabrowser.net/datapages/). From the NCBI GEO database (https://www.ncbi.nlm.nih.gov/geo/), two additional datasets of NSCLC samples were downloaded. This project gathered 1705 samples in total, including TCGA-LUAD (N=568), TCGA-LUSC (N=545), GSE68465 (N=462), and GSE4573 (N=130) datasets (**Table S1**). **Table S2** provides a summary of the clinical details of these samples. **Table S3** lists the clinical details of each sample from TCGA dataset. Among the 1579 NSCLC samples, survival status and survival time are available for 1558 samples. Transcripts per kilobase million (TPM) values were generated from FPKM values of RNA sequencing data downloaded from TCGA. The raw “CEL” files for the Affymetrix-produced GEO microarray data were downloaded. R packages “affy” and “simpleaffy” were employed to adjust the background and perform quantile normalization. The ComBat function from the “SVA” R package was used to remove the batch effect between TCGA and GEO datasets and the integrated data after removing the batch effect is provided in **Table S4**. The genomic mutation status of NSCLC patients from the TCGA database was displayed in an oncoplot generated with the R package “maftools”.

### Unsupervised consensus clustering of 25 m^6^A regulators

A total of 31 m^6^A regulators were gathered from the papers on m^6^A methylation modification. Due to the lacking of six m^6^A regulator genes (*IGF2BP1*, *KIAA1429*, *METTL16*, *METTL14*, *ALKBH5*, and *RBMX*) in GEO datasets, the remaining 25 m^6^A regulators were curated, including seven writers (METTL3, WTAP, RBM15, RBM15B, CBLL1, ZC3H13, and ZCCHC4), one eraser (FTO), 15 readers (YTHDF1, YTHDF2, YTHDF3, YTHDC1, YTHDC2, IGF2BP2, IGF2BP3, EIF3A, HNRNPA2B1, HNRNPC, FMR1, LRPPRC, ELAVL1, PRRC2A, and SND1), and two repellers (G3BP1 and G3BP2). Unsupervised consensus clustering was carried out using the expression levels of 25 m^6^A regulator genes to discriminate different m^6^A modification patterns with the R package “ConsensusClusterPlus”, which is based on a computational method called consensus clustering (20). Consensus Cumulative Distribution Function (CDF) and Delta area (relative change of area under the CDF curve) were used to select the proper clustering numbers within the high-throughput RNA-seq data. We use the parameters of a maximum evaluated k of 20, an 80% resampling rate, 1000 iterations, and Euclidean distance to determine the optimal number of clusters and to guarantee robustness.

### Gene set variation analysis (GSVA) and functional annotation

Using the R package “GSVA”, gene set variation analysis (GSVA), an unsupervised and non-parametric method, was used to compute the pathway enrichment scores in order to explore the biological process variations among different m^6^A modification patterns (21). To conduct GSVA analysis, the well-defined KEGG gene sets of “c2.cp.kegg.v6.2.symbols” were downloaded from the MSigDB database (https://www.gsea-msigdb.org/gsea/index.jsp). Gene set enrichment analysis with a cutoff value of false discovery rate (FDR) < 0.01 was used to examine biological processes correlated with m^6^A regulators using the R package “clusterProfiler”.

### Immune cell infiltration estimation

The single-sample gene-set enrichment analysis (ssGSEA) function from the R package “GSVA” was used to estimate the levels of immune cell infiltration. ssGSEA evaluates a specific gene set, including the gene expression data of 28 immune cells that represent different immune cell types, immune-related functions, and pathways in NSCLC (22). The enrichment scores representing the relative level of immune cell infiltration were compared between samples that belong to different m^6^A clusters by the Wilcox test. To illustrate their prognostic values, significantly different immune cells between m^6^A clusters were further analyzed by cox regression and visualized by the R package “forestplot”.

### Identification of differentially expressed genes (DEGs) between distinct m^6^A clusters

Differential expression analysis was carried out using the R package “limma”, an empirical Bayesian approach, to identify DEGs associated with m^6^A (23). Genes with adjusted *p *< 0.05 (Benjamini-Hochberg adjustment) and |fold change| > 1.5 in expression were regarded as DEGs. Hierarchical cluster analysis was used to divide NSCLC patients into genomic clusters based on the DEGs. We used a bottoms-up approach called agglomerative clustering, in which the data points were initially isolated as separate groups and then merged iteratively based on similarity until one cluster had been formed. The similarity was measured with Ward’s linkage, namely the Euclidean distance between two clusters was defined by the increase in the sum of squared after the clusters were merged.

### Dimension reduction and generation of m^6^A gene signatures

For all the identified differentially expressed genes between m^6^A clusters, the supervised machine learning algorithm random forest was applied for dimensionality reduction. After removing the redundant genes, the remaining genes more relevant to m^6^A modification went through survival analysis with the R package “Survminer”. Genes with significant survival results (*p *< 0.05) were added to a Cox regression model in further analysis. To explore the similarity between gene expression profile and prognosis efficiency, the m^6^A score was introduced. The m^6^A score was defined refer to the definition of gene expression grade index (GGI) (24), and the formula is as follows:


$$m^{6}Ascore\;=\;scale\left(\sum X-\sum Y\right)$$

Where scale represents the transformation parameter of standardization and X and Y are the expression of gene sets with positive and negative Cox coefficients, respectively. The optimal cutoff value was computed using the surv-cutpoint function from the “survival” R package. All samples were subsequently stratified into m^6^Ascore-high and m^6^Ascore-low subgroups, and their relationships with prognosis were evaluated as well.

### Correlation between m^6^A score and other pertinent biological processes

Mariathasan et al. have constructed a collection of genes to store genes related to a sort of biological processes, including Angiogenesis; CD8 T effector; Antigen processing machinery; Cell cycle; Cell cycle regulators; KEGG discovered histones; DNA damage repair; DNA replication; Fanconi anemia; FGFR3-related genes; Homologous recombination; Immune checkpoint; EMT1, EMT2, and EMT3 epithelial-mesenchymal transition (EMT) markers; Mismatch repair; Nucleotide excision repair; Pan-F-TBRS; WNT target (25). GSVA was used to quantify the above-mentioned biological processes in each sample with an enrichment score. Pearson correlation analysis was carried out between m^6^Ascore and enrichment score to reveal the relationship between m^6^Ascore and certain associated biological pathways.

### Copy number variation (CNV) analysis

The Genomic Identification of Significant Targets in Cancer (GISTIC) method was used to identify the common CNV regions across all samples with TCGA Copy Number Segment data. The significance threshold of GISTIC was: False Discovery Rate (FDR), namely q-value ≤ 0. 05. The peak region for each significant region was identified with a confidence interval of 0.95. The GISTIC analysis made use of the MutSigCV module from GenePattern, an online analysis tool provided by the Broad Institute (https://cloud.genepattern.org/gp/pages/index.jsf).

### Half maximal inhibitory concentration (IC_50_) prediction and tumor immune dysfunction and exclusion (TIDE) analysis

In order to predict the clinical chemotherapeutic response from tumor gene expression profiles, the IC_50_ values of clinical drugs (Cisplatin, Gemcitabine) were estimated using the R package “pRRophetic” (26). Then IC_50_ values between high m^6^Ascore samples and low m^6^Ascore samples were compared. In addition, signatures of T cell dysfunction and exclusion were analyzed using the online algorithm TIDE (http://tide.dfci.harvard.edu/) to predict the cancer immunotherapy response to immune checkpoint blockade (ICB) (27). A higher TIDE prediction score indicates a poor prognosis and a poor response to ICB therapy.

### NSCLC cell line m^6^A score calculation and chemotherapy drug IC_50_ validation

The Cancer Cell Line Encyclopedia was used to download the gene expression data for NSCLC cell lines (CCLE: https://sites.broadinstitute.org/ccle/). The m^6^A score for each cell line was calculated using the m^6^Ascore formula. NSCLC cell lines were stratified into m^6^Ascore-high and m^6^Ascore-low groups based on the cutoff value. Genomics of Drug Sensitivity in Cancer (GDSC: https://www.cancerrxgene.org/) provided information on the drug sensitivity of the chemotherapeutic drugs Cisplatin and Gemcitabine. In general, a dose titration of Cisplatin and Gemcitabine (6 nM-6 μM for Cisplatin; 0.1 nM-0.1 μM for Gemcitabine) was administered to STRs-verified cell lines for 72 hours in culture media after they had been seeded in 96-well plates and grown for 24 hours at 37°C in 5% CO_2_. Cell viability was determined using either a metabolic test (Resazurin or CellTiter-Glo) or DNA dye (Syto60). Every screening plate was put through rigorous quality control procedures. GraphPad Prism 9 software was used to calculate the IC_50_ of Cisplatin and Gemcitabine in NSCLC cell lines and to generate dose-response curves.

### Statistical analysis

The Wilcoxon test was used to determine whether scores between the two sample groups were statistically significant. The log-rank test from the R package “Survminer” was used to assess the statistical significance between the prognostic survival curves, which were generated using the Kaplan-Meier method. The prediction performance of immunotherapy by m^6^Ascore was assessed using the receiver operating character (ROC) curve, and the area under the ROC curve (AUC) was computed using the R package “pROC”. Patients with high and low m^6^Ascores had different mutational landscapes, which were visualized using the “maftools” R package.

## Results

### Genetic and transcriptional alteration landscapes of four types of RNA m^6^A methylation regulators in NSCLC

**Figure 1:**
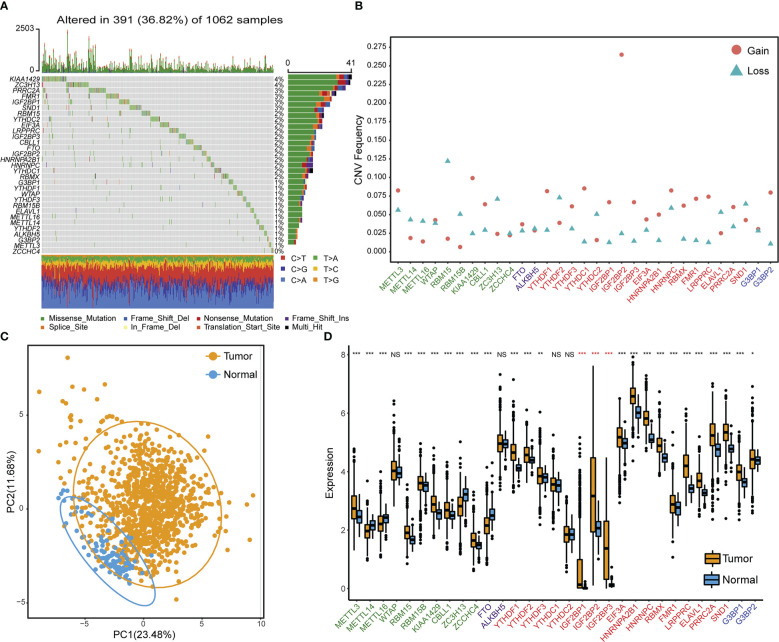
Genetic and transcriptional variation landscapes of RNA m^6^A modification regulator genes in the TCGA dataset. **(A)** The frequency of mutations and the distribution of mutation types of m^6^A regulator genes in NSCLC tissue samples. **(B)** The frequency of m^6^A regulator gene copy number variation in NSCLC tissue samples. Blue triangles denote deletion and red dots denote amplification. **(C)** Principle component analysis results to distinguish NSCLC samples from adjacent normal tissues based on the expression of m^6^A regulator genes. **(D)** The expression profile of m^6^A regulator genes in cancer samples and adjacent normal samples. Asterisks in red indicate genes belonging to the IGF2BPs family. NS *p* > 0.05; *p < 0.05; **p < 0.01; ***p < 0.001.

**Table S5** lists the 31 RNA m^6^A modification regulators that were used in this study, which included ten methyltransferases “writers”, two demethylases “Erasers”, 17 RNA binding proteins “Readers”, and two “Repellers”. We first summarized the occurrence frequency of somatic mutations and copy number variations in 31 m^6^A regulator genes in TCGA NSCLC samples. Specifically, *ZC3H13* and *KIAA1429* had the greatest mutation frequency, reaching 4%. The most frequent mutation type was missense mutation (**Figure 1A**). Copy number variation (CNV) frequency analysis showed that copy numbers were generally changed among 31 regulatory factors. Copy number amplification commonly occurred in genes such as *IGF2BP2*, *KIAA1429*, and *YTHDC1*, while copy number deletion commonly occurred in genes such as *RBM15*, *YTHDF2*, and *ZC3H13* (**Figure 1B**, **Table S6**). According to the expression of these 31 m^6^A regulator genes, principle component analysis could differentiate TCGA NSCLC samples from adjacent normal samples (**Figure 1C**). Gene expression analysis of 31 m^6^A regulators between TCGA NSCLC samples and adjacent control samples showed that most regulator genes were significantly overexpressed in NSCLC tissues, especially reader genes (*IGF2BP1*, *IGF2BP2*, and *IGF2BP3*) from the IGF2BPs family (**Figure 1D**).

### Unsupervised clustering of m^6^A regulator genes

**Figure 2:**
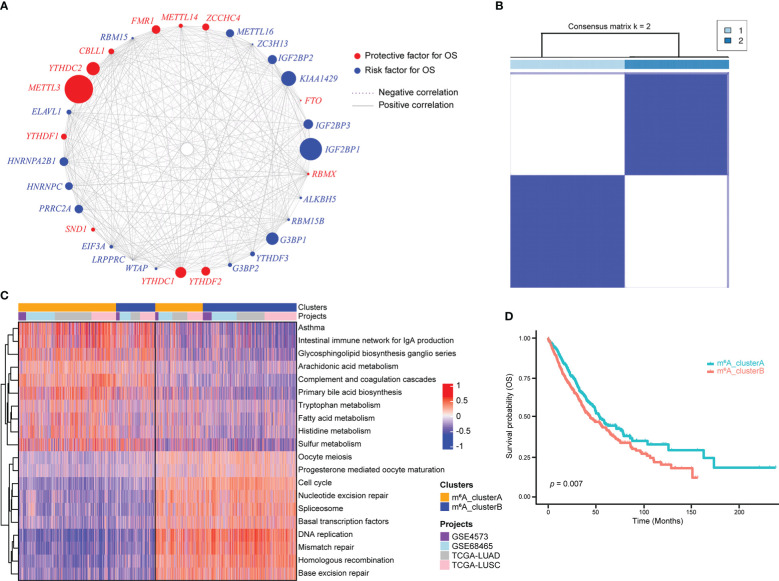
m^6^A regulator genes unsupervised clustering in NSCLC samples. **(A)** m^6^A regulator gene interactions in NSCLC. The circle size reflects how each gene affects the ability to predict survival. The stronger the association between gene expression and prognosis, the larger the circle. The red circle indicates prognostic protective factors and the blue circle indicates prognostic risk factors. The spearman correlations between genes are linked by lines connecting them, with positive correlations denoted by solid grey lines and negative correlations by purple dotted lines. The interaction strength between m^6^A regulators determines line thickness. **(B)** Consensus clustering of m^6^A regulator genes, 1 and 2 represent two subgroups. **(C)** GSVA enrichment analysis revealed the activation state of biological pathways with distinct m^6^A clusters. The heatmap was used to depict these biological processes, with red indicating activation and blue indicating inhibition. **(D)** Two m^6^A clusters showed a significant survival differences, as depicted in the Kaplan-Meier curve.

An m^6^A regulatory network was constructed for 1557 NSCLC samples with expression data and survival information available to describe the spearman correlations within m^6^A regulator genes and the correlations between m^6^A regulator genes and NSCLC prognosis (**Figure 2A**). Results suggested that different m^6^A modification patterns might be significantly influenced by interactions between different functional types of m^6^A regulators. Due to the absence of *IGF2BP1*, *KIAA1429*, *METTL16*, *METTL14*, *ALKBH5*, and *RBMX* expression data in GEO datasets, the remaining 25 m^6^A regulator genes were included for consensus clustering. Two subgroups were identified using unsupervised consensus clustering, and their respective names were m^6^A_clusterA and m^6^A_clusterB (**Figure 2B**). Biological pathway differences between two m^6^A clusters were identified using GSVA enrichment analysis. m^6^A_clusterA significantly enriched metabolic-related biological processes like fatty acid metabolism and tryptophan metabolism, while m^6^A_clusterB considerably enriched replication- and transcription-related biological pathways such as DNA repair and mismatch repair (**Figure 2C**, **Table S7**). The two m^6^A clusters also had significantly different prognoses, as demonstrated by the Kaplan-Meier curve of overall survival (*p* = 0.007) (**Figure 2D**).

**Figure 3:**
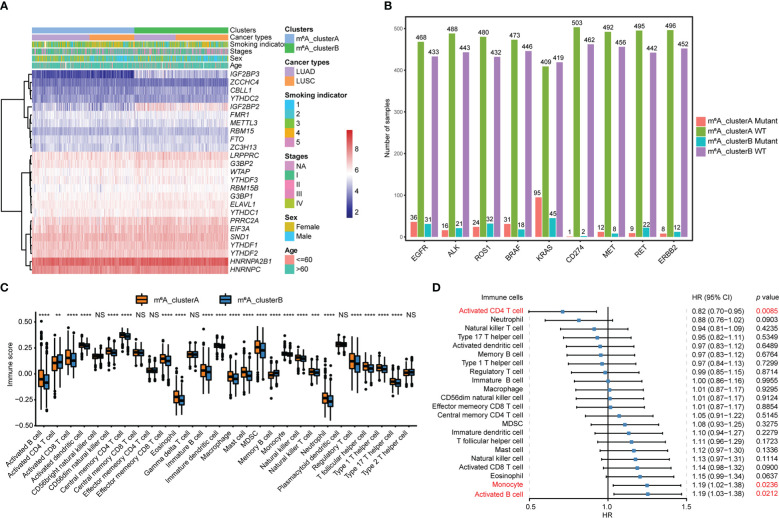
Transcriptome characteristics and immune cell infiltration features in two m^6^A regulator gene clusters. **(A)** The expression pattern of the m^6^A regulator genes between the two m^6^A clusters that matched the clinical information, including cancer type, smoking indicator, stage, sex, and age. **(B)** The nine most common gene mutation statuses in NSCLC. **(C)** The boxplot shows the abundance of 28 infiltrating immune cells among the two m^6^A clusters. NS *p* > 0.05; **p < 0.01; ***p < 0.001; ****p < 0.0001. **(D)** Forest plots of hazard ratios (HRs) for 22 immune infiltration cells associated with OS and meaningful immune cells were marked in red (*p *< 0.05).

The heatmap of 25 m^6^A regulator genes, which was classified by two m^6^A clusters, showed the relationship between the expression level and matching clinical information, such as cancer type, smoking indicator, stage, sex, and age. The distribution of clinical information between the two m^6^A clusters does not significantly differ. Notably, NSCLC patients in m^6^A_clusterB were more likely to express the IGF2BPs family, including *IGF2BP2* and *IGF2BP3* (**Figure 3A**). Among the most common gene mutations in people with NSCLC, we analyzed the mutation status of nine genes: *EGFR*, *ALK*, *ROS1*, *BRAF*, *KRAS*, *CD274*, *MET*, *RET*, and *ERBB2*. Results showed that only the mutation status of *KRAS* (*p* = 7.74e-05) and *RET* (*p* = 0.015) were significantly different between m^6^A_clusterA and m^6^A_clusterB, while the other seven genes were not significant (Chi-square test, *p* > 0.05). Excluding *KRAS*, NSCLC samples without the above-mentioned gene mutations are more in m^6^A_clusterA than in m^6^A_clusterB (**Figure 3B**). Furthermore, to illustrate the impact of m^6^A regulators on immune cell infiltration, ssGSEA was conducted based on the sample expression data and obtained the proportion distribution of 28 immune cell types in two different m^6^A clusters of NSCLC samples (**Figure 3C**). Results showed that 22 of 28 immune infiltration cells had differential expression between the two m^6^A clusters and most of them were highly expressed in m^6^A_clusterA, except for activated CD4 T cells and memory B cells, which were highly expressed in m^6^A_clusterB (**Figure 3C**, **Table S8**). This suggests that m^6^A_clusterA has an immune microenvironment that is hot and suppressive. For the 22 differentially expressed immune infiltration cells, univariate Cox regression analysis revealed that activated CD4 T cells (*p* = 0.0085), monocytes (*p* = 0.024), and activated B cells (*p* = 0.0212) were significantly associated with the prognosis of two m^6^A clusters (**Figure 3D**, **Table S9**).

### Identification of m^6^A signature genes

**Figure 4:**
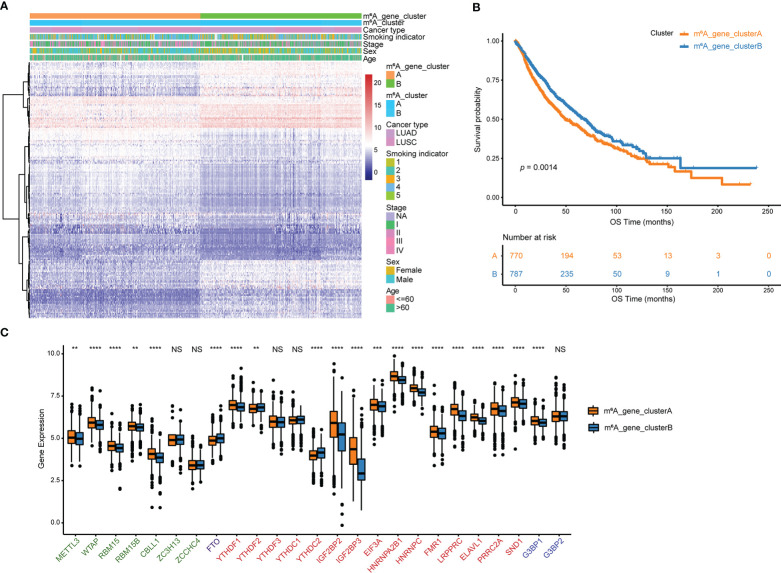
Genes that differ in expression are associated with m^6^A. **(A)** Unsupervised analysis and hierarchical clustering of differentially expressed genes associated with the m^6^A phenotype to divide NSCLC patients into two genomic groups.Patient annotations include m^6^A cluster, cancer type, smoking indicator, stage, sex, and age. **(B)** Kaplan-Meier curve depicts the association between m^6^A modification genomic phenotypes and OS. **(C)** The two m^6^A gene clusters’ expression of 25 m^6^A regulator genes. NS *p* > 0.05; **p < 0.01; ***p < 0.001; ****p < 0.0001.

Using the R package “limma”, 194 m^6^A phenotype-related differentially expressed genes between m^6^A clusters were identified in order to explore the probable biological functions of each m^6^Acluster (**Table S10**). Unsupervised hierarchical cluster analysis has classified the NSCLC patients into two genomic clusters, termed m^6^A_gene_clusterA and m^6^A_gene_clusterB, which are roughly in accordance with the m^6^A modification pattern, based on the expression of the 194 differentially expressed genes (**Figure 4A**, **Table S11**). The log-rank test and Kaplan-Meier curve indicate that the genomic phenotypes of m^6^A modification were significantly related to OS in NSCLC patients and patients in m^6^A_gene_clusterB had better prognoses (**Figure 4B**, *p* = 0.0014). Among the 25 m^6^A regulator genes, 17 were significantly more abundantly expressed in m^6^A_gene_clusterA than in m^6^A_gene_clusterB, while three were significantly highly expressed in m^6^A_gene_clusterB (**Figure 4C**).

### Clinical and transcriptome features of m^6^A-related phenotypes

**Figure 5:**
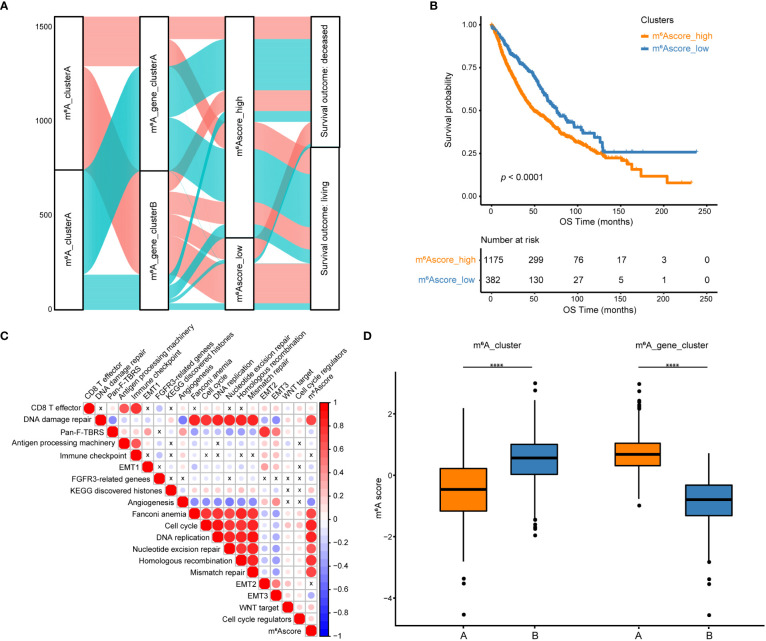
Construction of m^6^A scores. **(A)** An alluvial diagram of the m^6^A clusters showing different m^6^A gene clusters, m^6^Ascores, and survival outcomes. **(B)** The Kaplan-Meier curve demonstrated a significant correlation between m^6^A scores in the high and low groups and overall survival. **(C)** GSVA and Pearson correlation analysis show correlations between m^6^Ascore and biological pathways constructed with known gene signatures in NSCLC. A positive correlation is indicated by red, while a negative correlation is indicated by blue. The circle size is inversely proportional to the levels of significance, and X indicates no significant correlation. **(D)** Boxplots show the differences in m^6^A scores between m^6^A clusters and between m^6^A gene clusters. ****p < 0.0001.

Among the 194 differentially expressed genes derived from the previous analysis, redundant genes were removed using the random forest algorithm, leaving 83 feature genes that were most closely related to the m^6^A relationship between these genes and NSCLC patient survival. These 83 genes were classified into two groups, a positive group with 30 genes and a negative group with 53 genes, based on the coefficient value of genes obtained from the Cox regression (**Table S12**). According to the m^6^Ascore formula, m^6^A scores for all samples were calculated and the best cutoff of m^6^Ascore (cutoff = -0. 7437558) was established by the “surv_cutpoint” function of the R package to classify NSCLC samples into m^6^Ascore high and low groups (**Figure 5A**, **Table S13**). Results of the survival analysis demonstrated that the m^6^Ascore might accurately characterize the prognosis of NSCLC patients (*p *< 0.0001), with the m^6^Ascore_low group has a good prognosis while the m^6^Ascore_high group has a bad prognosis (**Figure 5B**). To better depict the function of m^6^Ascore, GSVA was performed with known gene signatures. A significant positive correlation between m^6^Ascore and biological processes, including the cell cycle and DNA replication, has been found using pearson correlation analysis. In contrast, the correlation between m^6^Ascore and other biological processes, such as angiogenesis and EMT3, is significantly negative (**Figure 5C**, **Table S14**). m^6^Ascores between m^6^A clusters and between m^6^A gene clusters were compared using the Wilcox test. Results showed that m^6^A_clusterA had a substantially lower m^6^Ascore than m^6^A_clusterB, while m^6^A_gene_clusterA had a significantly higher m^6^Ascore than m^6^A_gene_clusterB (**Figure 5D**). Furthermore, as shown in **Figure S2**, there are significant differences in m^6^Ascores across several clinical categories, including EGFR mutation status, age, and sex (**Figures S2A, B**). Additionally, in both the TCGA and GEO datasets, NSCLC patients with high and low m^6^Ascores had significantly different overall survival probabilities (**Figure S2C**).

### Molecular characteristics of m^6^Ascore groups in TCGA datasets

**Figure 6:**
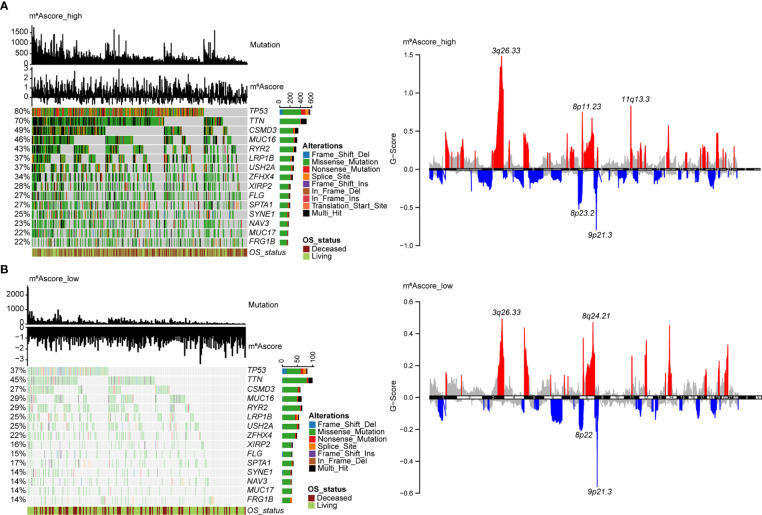
Molecular features of m^6^Ascore groups in TCGA datasets. **(A)** The distribution of tumor somatic mutations in NSCLC with a high m^6^Ascore is depicted in the waterfall plot. Each column represents a patient. TMB is depicted in the upper barplot, and each gene’s mutation frequency is indicated by the number on the left. The horizontal barplot on the right depicts the percentage of each variant type, and overall survival status is shown as patient annotations (left). GISTIC plot depicts the distribution of somatic copy number alterations in NSCLC with a high m^6^Ascore, regions of copy number amplification are highlighted in red, and regions of copy number deletion are highlighted in blue. Several significant gene names are marked (right). **(B)** The waterfall plot displays the somatic mutation distribution (left), and the GISTIC plot shows the distribution of somatic copy number alterations (right) in NSCLC with a low m^6^Ascore.

The distribution of tumor somatic mutations in TCGA NSCLC datasets is visualized by the R package “maftools” (**Figures 6A, B**, left), and the GISTIC algorithm was used to evaluate and visualize the distribution of somatic copy number alterations in groups with high and low m^6^Ascores, respectively (**Figures 6A, B**, right). The mutational landscape revealed that the top 15 genes in the high m^6^Ascore group had higher tumor mutation frequencies than those in the low m^6^Ascore group. The most mutational gene was *TP53* (80% vs. 37%). GISTIC plots revealed that the m^6^Ascore low group had significantly fewer copy number alterations than the m^6^Ascore high group, which is consistent with somatic mutations. Based on these data, we were able to more fully illustrate the impact that m^6^A score classification has on genomic variation and to demonstrate the potentially intricate interactions between individual somatic mutations/alterations and m^6^A modifications.

### The value of m^6^Ascore in predicting chemotherapy and immunotherapy response

**Figure 7:**
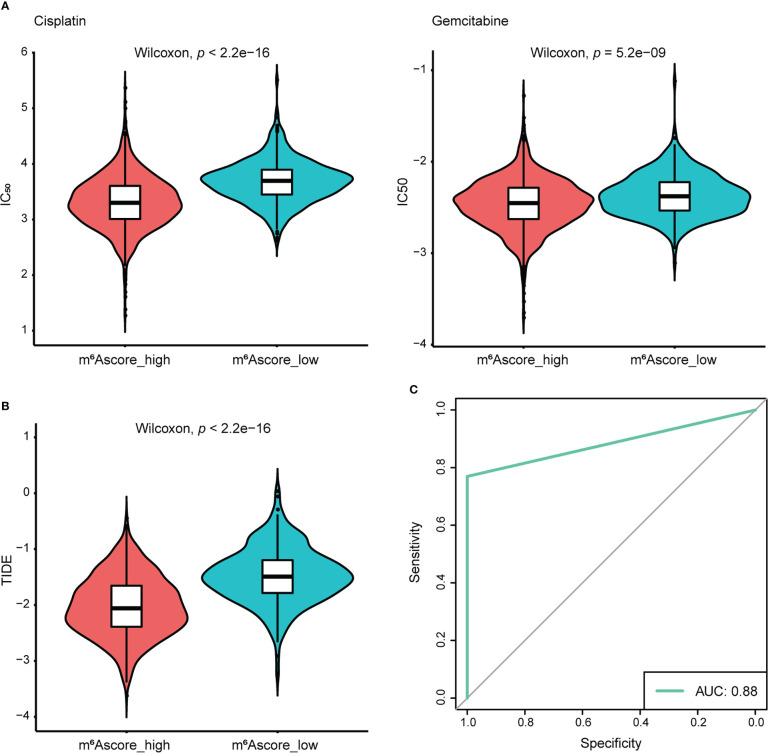
The response of the high and low m^6^Ascore groups to chemotherapy/immunotherapy. **(A)** Differences in IC_50_ of Cisplatin and Gemcitabine treatment. **(B)** Differences in TIDE prediction score. **(C)** The receiver operator characteristic curve (ROC) shows the predictive performance of m^6^Ascore in NSCLC patients receiving anti-PD1 and anti-CTLA4 therapy (AUC, 0.88).

To extend the potential therapeutic use of m^6^Ascore, we explored whether the intrinsic m^6^Ascore in cancer cells could predict their response to various drugs, which was inspired by the cross-talk between m^6^Ascore and many key cancer-related pathways. Using the R package “pRRophetic” and the expression profile from TCGA and GEO datasets, IC_50_ values of chemotherapeutic drugs Cisplatin and Gemcitabine were calculated. According to a comparison of the relative distribution of Cisplatin and Gemcitabine IC_50_ values, the IC_50_ value in the low m^6^A score group was significantly higher than that in the high m^6^A score group, indicating that the high m^6^A score group had poor drug resistance (**Figure 7A**). Moreover, based on the mRNA expression profile in the TCGA data set, the TIDE algorithm was employed to assess the clinical effect of ICB treatment in the m^6^Ascore high and low groups. Results revealed that patients with high m^6^Ascores had TIDE scores that were significantly lower than those with low m^6^Ascores. That was to say, compared to patients with low m^6^Ascores, NSCLC patients with high m^6^Ascores exhibited a better therapeutic benefit and clinical response to anti-PD1 or anti-CTLA4 immunotherapy (**Figure 7B**). According to the ROC curve, the m^6^Ascore may be a reliable biomarker for predicting outcomes and evaluating the therapeutic efficacy of anti-PD1 and anti-CTLA4 treatments (AUC = 0.88) (**Figure 7C**).

**Figure 8:**
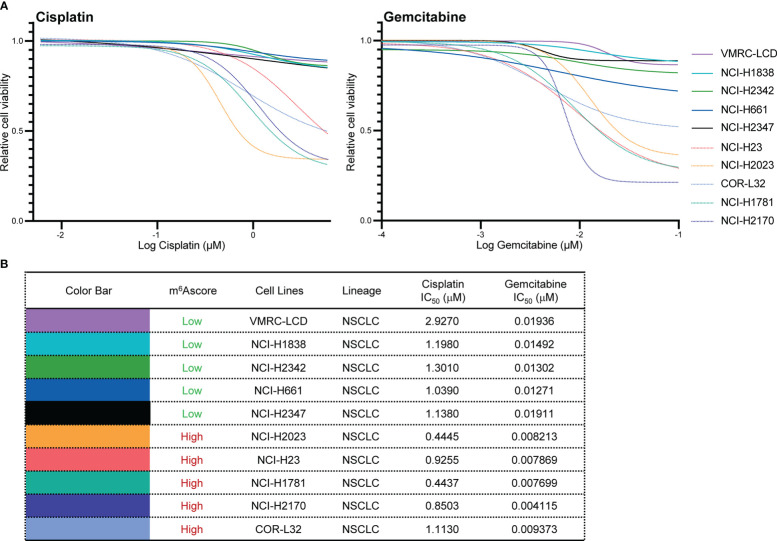
Validation of chemotherapy drugs’ IC_50_. **(A, B)** Measurement of IC_50_ by DNA dye or metabolic assay in NSCLC cell panels (VMRC-LCD, NCI-H1838, NCI-H2342, NCI-H661, NCI-H2347, NCI-H23, NCI-H2023, COR-L32, NCI-H1781, NCI-H2170) treated with Cisplatin and Gemcitabine.

To validate the chemotherapy response in high and low m^6^Ascore groups, we first classified NSCLC cells into m^6^Ascore-high (NCI-H23, NCI-H2023, COR-L32, NCI-H1781, and NCI-H2170) and m^6^Ascore-low (VMRC-LCD, NCI-H1838, NCI-H2342, NCI-H661, and NCI-H2347) groups with distinct differences. The dose-response curves and IC_50_ values indicated that Gemcitabine treatment is generally more effective in NSCLC cell lines. The m^6^Ascore-high group of NSCLC cell lines has higher anti-tumor activity when treated with Cisplatin and Gemcitabine (**Figures 8A, B**), which is in line with the predicted result that the group with a high m^6^A score has poor drug resistance.

## Discussion

The immunological state of TME in diverse malignancies is regulated by RNA m^6^A modification (28, 29). Recent studies have uncovered the relationships between m^6^A regulators and immune cell infiltration. The accumulation of myeloid-derived suppressor cells necessary to maintain the activation and proliferation of CD4+ and CD8+ T cells was reduced when *METTL3* was selectively depleted in colorectal cancer cells (30). Natural killer cell homeostasis and maturation, as well as their anti-tumor and antiviral activity, are positively regulated by YTHDF2 (31). *YTHDF1* deficiency in classical dendritic cells could enhance antigen presentation, initiate anti-tumor responses, and improve the therapeutic effectiveness of PD-L1 checkpoint blockade (32). Nevertheless, these research concentrated on the single m^6^A modification regulators, and the integrated roles of various m^6^A regulators in modifying immune characteristics in NSCLC need to be further investigated. More efficient immunotherapy approaches will result from a knowledge of the involvement of distinct m^6^A modification patterns in TME cell infiltration and the TME anti-tumor immune response.

The global transcriptional and genetic profiles of m^6^A modification regulator genes and their mutual correlation in NSCLC were the focus of this study. When compared to healthy controls, the expression of *METTL3* and *METTL14* in NSCLC samples exhibited a contrary trend. As well-studied m^6^A writers, METTL3 and METTL14 are reasonably thought to have similar functions. Although a similar phenomenon exists in prostate cancer (33), further studies are required in the future to figure this out. m^6^A readers from the IGF2BPs family showed high expression levels and mutation frequency, especially *IGF2BP2*. According to Li et al., IGF2BP2 and IGF2BP3 was essential for lung cancer progression, and they could identify and stabilize m^6^A sites and function as ‘readers’ in the post-transcriptional regulation manner (34). A study found that IGF2BP2 regulated macrophage activation in an m^6^A-dependent manner, which indicated a potential therapeutic target of macrophages in inflammatory diseases (35). RBM15 and its paralogue RBM15B contain RNA-binding motifs, which make it easier to recruit m^6^A methyltransferase to specific sites in RNA (36, 37). Our results showed that *RBM15* and *RBM15B* were significantly up-regulated in NSCLC samples and had frequent CNV alterations, indicating their potential role in promoting cancer cell migration and invasion (38). Our comprehension of how epigenetic regulation affects diverse physiological processes and TME cell-infiltrating characterization will be enhanced by a thorough evaluation of the patterns of m^6^A modification, which will highlight the heterogeneity of m^6^A modification. These regulators’ effects on immune infiltration mechanisms *via* m^6^A modification require additional biology studies utilizing cell culture and even PDX mice models.

With consistent clustering, we have identified two independent m^6^A modification patterns with significantly different TME immune cell infiltration traits, among which m^6^A_clusterA showed immune inflamed phenotype and enriched abundant immune cells, known as hot tumor. Immune checkpoint inhibitors are frequently associated with greater benefits in such tumors (39). Therefore, m^6^A_clusterA had a better prognosis. As defined by us, m^6^A signature genes are the genes that are expressed differently in different m^6^A modification patterns. Two genomic clusters were identified using m^6^A signature genes, which is consistent with m^6^A modification clusters. m^6^A regulator genes were also differentially expressed between genomic clusters. This phenomenon demonstrates once more how the m^6^A modification significantly shapes various TME landscapes. Taking into account the individual variability of m^6^A modification, an m^6^Ascore algorithm based on the m^6^A signature genes was developed to compute the m^6^Ascore of each sample. m^6^Agenecluster and m^6^Ascore groups have apparent differences in prognosis, clinical features, or molecular characteristics. As a result, we demonstrated that m^6^Ascore might be used to evaluate the clinicopathological traits of patients, such as tumor inflammation, prognosis, genetic variation, and so forth. This demonstrated that m^6^Ascore was robust and reliable and could be utilized to identify the tumor immune phenotypes by comprehensively assessing individual tumor m^6^A modification patterns. Therefore, m^6^Ascore could be utilized to predict the effectiveness of chemotherapy and the patient’s clinical response to anti-PD1 and anti-CTLA4 immunotherapy for NSCLC, in addition to being used as an independent prognostic biomarker to predict patients’ survival. Immunotherapy response results predicted from m^6^Ascore classification demonstrated that the predictive performance of the m^6^Ascore in NSCLC patients treated with drugs anti-PD1 and anti-CTLA4 reaches a meaningful result (AUC = 0.88). Several novel ideas for cancer immunotherapy that alter m^6^A modification patterns by targeting m^6^A regulators or m^6^A signature genes were presented in our study. By harnessing the immune system, firing up the TME cell infiltration characterization to turn tumors from “cold” to “hot” is a promising strategy to explore novel drug combinations or novel immunotherapeutic agents.

This study is the first to comprehensively and methodically analyze the relationships between m^6^A regulator modification patterns, immune infiltration, and treatment resistance in NSCLC. Their relationships attracted emerging attention in recent years (40). The numerous results generated from this study will provide hints for other researchers in deep mechanism study and direct m^6^A immunotherapy in lung cancer. In addition, in the analysis process, our study is the first to use public drug sensitivity experimental data to validate the value of m^6^Ascore in predicting chemotherapy response, making our prediction results more reliable. However, limitations exist in this study. Firstly, when merging two large TCGA cohorts and two GEO datasets of NSCLC patients, intra-tumor heterogeneity in different databases was not considered. A study has proven that tumor heterogeneity affects cancer immunotherapy (41). Secondly, this study confirmed the strong impact of m^6^A modification on the immune characteristics of NSCLC, but only theoretically valid. Bench works are warranted in the future to explore the underlying mechanisms. Thirdly, this study lacks an external clinical cohort to verify the results. Therefore, large external NSCLC cohorts are needed for further validation.

## Conclusions

In this study, a thorough examination of m^6^A regulators in NSCLC was conducted with bioinformatics analysis. We initially screened DEGs of m^6^Aclusters, then separated NSCLC patients into two categories, and computed the m^6^Ascore in order to build a risk model with a good predictive value for prognosis. The results of this study may help us learn more about how m^6^A signaling influences the progression and prognosis of NSCLC. This work emphasizes the important clinical implications of RNA modifications’ cross-talks and contributes to developing individualized immune therapeutic approaches for NSCLC patients.

## Data availability statement

The datasets presented in this study can be found in online repositories. The names of the repository/repositories and accession number(s) can be found in the article/**Supplementary Material**.

## Author contributions

YW and BY conceived and designed this study. BY performed the analysis, interpreted the data, and wrote the manuscript. HQ, MZ, YY, XT, HY, and WH helped review the data and manuscript. YW supervised the study. All authors contributed to the article and approved the submitted version.
