# Supplementary material for: m6A regulators featured by tumor immune microenvironment landscapes and correlated with immunotherapy in non-small cell lung cancer (NSCLC)
Source: Front Oncol. 2022 Dec 16;12:1087753. doi: 10.3389/fonc.2022.1087753 (PMC9800857; doi:10.3389/fonc.2022.1087753)
Supplement: Supplementary file 1 [file Image_1.pdf]

Supplementary Material

Supplementary Figures

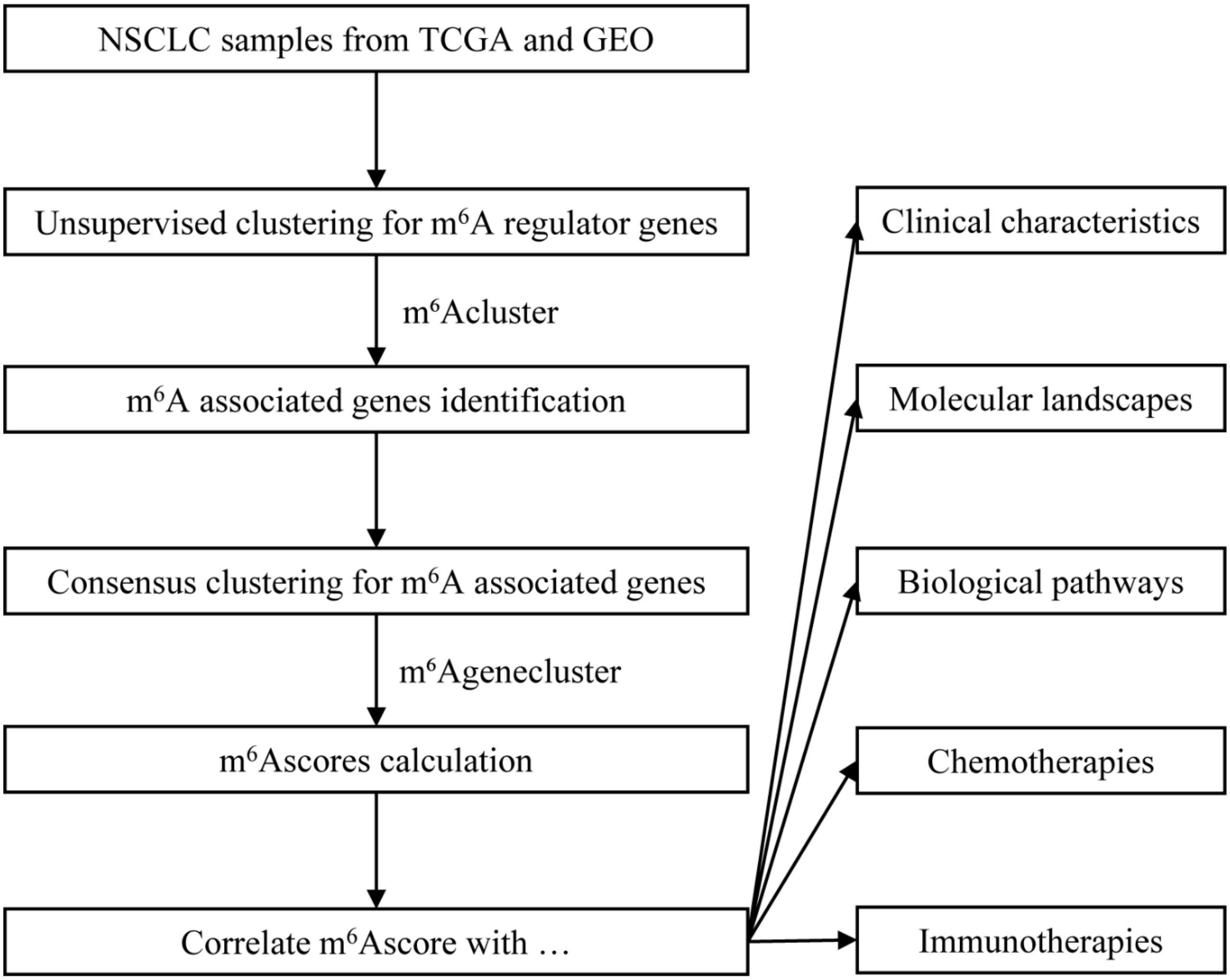

Supplementary Figure 1. An overview of the workflow of this study.

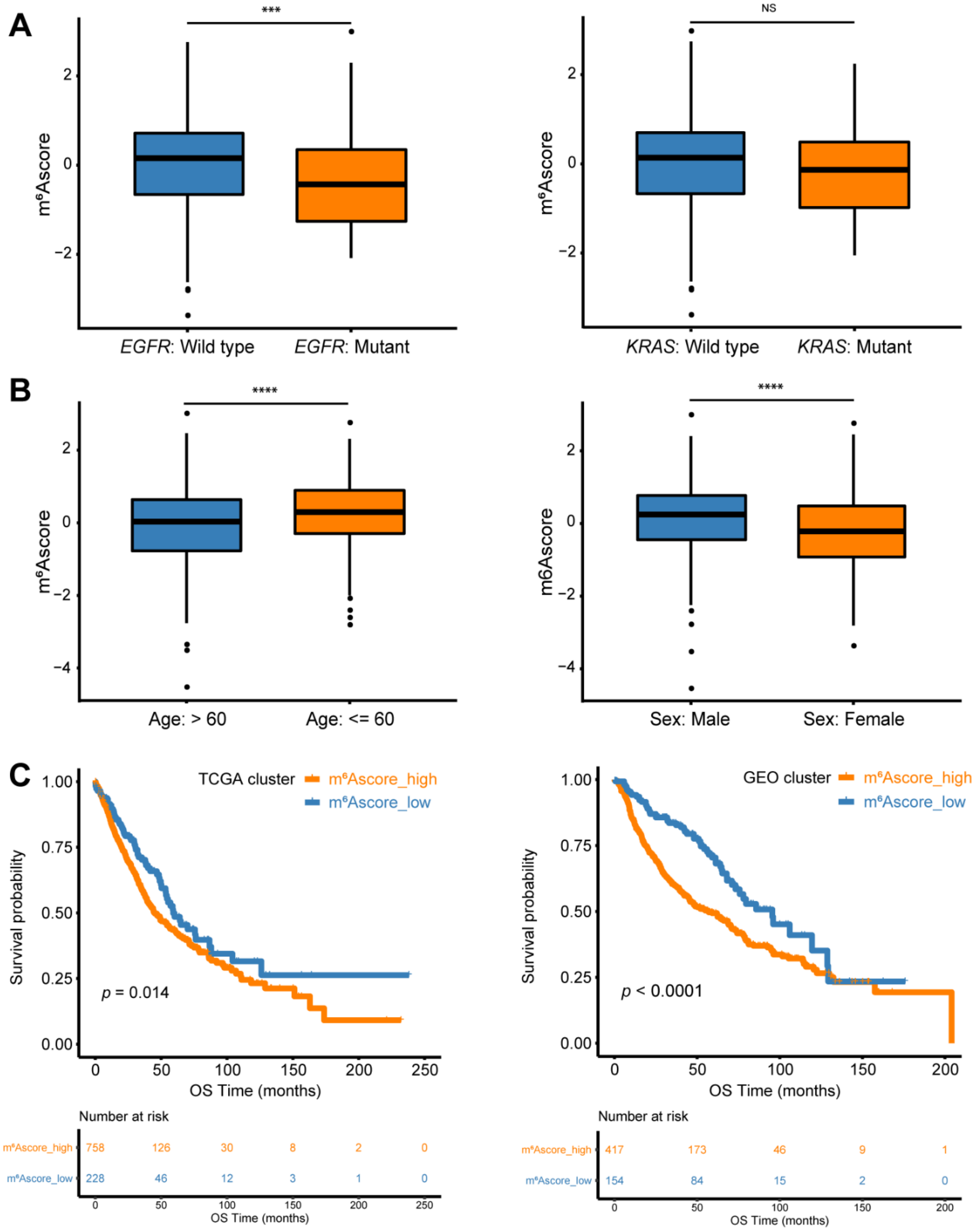

Supplementary Figure 2. Comparison of m^{6}Ascores between different groups. (A) The distribution of m^{6}Ascores in NSCLC patients with EGFR or KRAS mutations. (B) The distribution of m^{6}Ascores by age or sex in NSCLC patients. (C) The overall survival probability of NSCLC patients in the high and low m^{6}Ascore groups from TCGA and GEO datasets, respectively.
